# BlockEdge: A Privacy-Aware Secured Edge Computing Framework Using Blockchain for Industry 4.0

**DOI:** 10.3390/s23052502

**Published:** 2023-02-23

**Authors:** Deepsubhra Guha Roy

**Affiliations:** Department of Internet of Things, School of Computer Science and Engineering, Vellore Institute of Technology, Vellore 632014, India; roysubhraguha@gmail.com or deepsubhra.guha@vit.ac.in

**Keywords:** security threat, edge computing, distributed ledger, blockchain, digital signature, Industry 4.0

## Abstract

Edge computing has its application in a lot of areas now, but with the increasing popularity and benefits, it suffers from some challenges such as data privacy and security. Intruder attacks should be prevented and only authentic users should have access to data storage. Most of the authentication techniques apply some trusted entity to undergo the process. Users and servers both have to be registered in the trusted entity to get permission of authenticating other users. In this scenario, the entire system depends on a single trusted entity; so, a single point of failure can cause the failure of the total system, and scalability issues are there also. To address these issues remaining in the existing systems, in this paper, a decentralized approach has been discussed which is capable of eliminating the concept of a single trusted entity by introducing a blockchain paradigm in edge computing where every time a user or server wants to enter the system, it does not have to register itself manually, but the authentication process is carried out throughout the scheme automatically. Experimental results and performance analysis prove that the proposed architecture is definitely beneficial and it outperforms the existing ones in the concerned domain.

## 1. Introduction

Cloud computing and its associated technologies are some emerging paradigms in today’s world as they have proved their efficiency in having irresistible demand for pervasive intelligence in various fields. Despite having different benefits, the cloud still suffers from challenges such as resource centralization leading to end users’ separation from their corresponding cloud service which results in latency increase and jitter ultimately. They have become a major issue as the requirements of end users are dynamic mostly, and the introduction of ubiquitous network architecture took place just due to this. Big data processing components include data mining, data acquisition, pre-processing, and pattern recognition using real-time applications such as healthcare monitoring and control, GPS navigation, Internet of Things (IoT) where low latency is highly desired to satisfy the end-user requirements.

Social networks, smart cities, smart grids, Internet of Everything (IoE) are the main areas of Industry 4.0 which benefited a lot from the cloud paradigm and face different challenges as well. Jitter and latency have been two major issues as the user requirements keep changing always. To deal with these situations, ubiquitous computing came into the picture and proved its efficiency in its concerned domain, sometimes by introducing a content delivery network (CDN) which is all about web content caching along with computation accompanying it. This work is concerned with several shortcomings found in the conventional centralized architecture of cloud computing platforms in Industry 4.0, which can be summarized as follows:

- Multi-source data processing: The requirements of multi-source data processing cannot be met by the linearly growing capability of the cloud at the network edge.
- Data transfer speed: The speed of data transfer and bandwidth suffer from a bottleneck as long-distance interactions result in latency and computing resource wastage.
- Privacy and security: During the transmission, privacy and security can become major issues for edge device users.

### Motivation and Contribution

While scientists are working on overcoming these issues, there are several security challenges that remain unaddressed till now. In this paper, privacy preservation and data security have been attempted to be achieved. The authentication schemes have been implemented using an extended decentralized form contributing to the following factors.

- Addressing the gaps in conventional architectures: After reviewing recent research works and their loopholes in the concerned field, the blockchain mechanism has been integrated with edge computing and the expected challenges of this task have been evaluated also.
- Use of permission blockchain: Among two categories of blockchain, i.e., permissioned and permissionless, the principle of the first one has been followed during the deployment of the comprehensive decentralized scheme.

**Figure 1:**
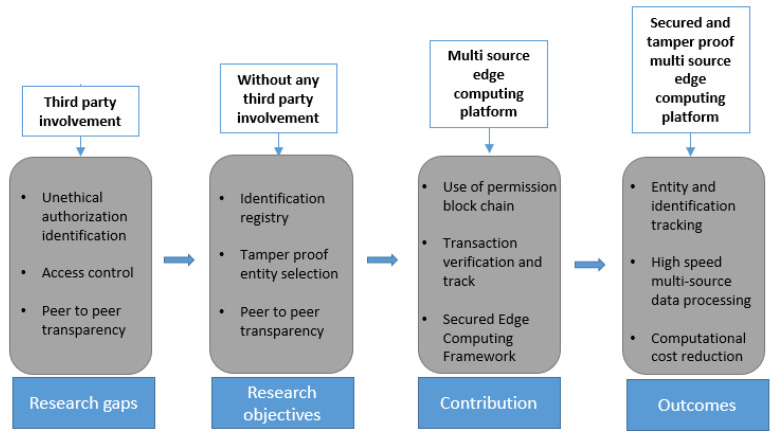
Contribution of the proposed system.

In brief, the proposed work contributes to the following outcomes in the field of edge computing, also shown in Figure 1.

- Track each transaction and entity selection through identification and authorization.
- Access control privacy mechanism while using data transfer in image format from the central cloud to the edges.
- Peer-to-peer transparency and tamper-proofing mechanism through blockchain help increase data transfer speed.
- Reduction of third-party involvement cut off the computational cost of multi-source data processing.

The next section analyzes different related studies along with their benefits and drawbacks. The motivation behind introducing this proposed method is explained which is actually overcoming the loopholes found in the existing literature and the major contributions are discussed also. This proposed methodology is presented in Section 3 and the experimental setup along with the performance analysis of the outcome is discussed in Section 4. The concluding statements and probable future directions of this research are discussed in Section 5.

## 2. Literature Survey

Mobile edge computing suffers from different security threats which differ from the cloud computing paradigm because of the additional features of edge computing namely heterogeneous architecture [1], location awareness, and mobility support. In each network tier, the threats should be addressed to protect the end user data. To prevent intruders from accessing, misusing, and abusing resources, an access control mechanism should be introduced where a unique identifier can be assigned to each and every entity in the network for better authentication in the system.

### 2.1. Challenges in Edge Platform

There are different forms of edge computing, i.e., fog computing [2,3,4,5], mobile cloud computing [6,7,8], mobile edge computing [9,10], dew computing [11], and others; Table 1 has differentiated them by using some parameters, i.e., deploying infrastructure, ownership, deployment platform, purpose, and target users. However, all of them have the same aim to bring cloud services to the network edge so that the distance from the cloud infrastructure to the end user device gets more reduced than before for better service quality by improving response time.

If the network core is replicated at the edge of the network, computation offloading gets easier and network burden is reduced by preserving privacy and security also.

### 2.2. Functionality of Blockchain

Among two types of the blockchain network, the permissionless blockchain cannot control the entry of users in the network; thus, every node in the network is bound to participate in a broad consensus for getting verified. The consensus protocols are stricter in this case for avoiding conspiracies in the network, e.g., bitcoin applies Proof of Work (PoW) consensus for this purpose. On the other hand, permissioned blockchain has a control over the entries in a network consisting of some limited users participating in the consensus and others as observers. All the nodes are detectable within this system and regulated using simpler algorithm and having lesser intensity than the public network.

### 2.3. Characteristics

Blockchain can be characterized by the following points based on some principles followed by it to get more adopted in various systems. The attributes include:

#### 2.3.1. Peer-to-Peer

Trusted third party (TTP) needs to exist in the network during the transaction, otherwise every node gets the same privilege and right to connect with each other. The network duties and controls are circulated among peers to increase the overall security in the system [12].

#### 2.3.2. Transaction Verification

Integrity and authenticity of the ongoing transactions are verified and maintained by every node participating in the task as miner or observer which, in turn, leads to an increased trust in the network and a transaction gets approved only after getting permission from the maximum of the nodes.

#### 2.3.3. Transparency

The initial transaction in the system is responsible for checking and auditing each and every transaction where all the users are allowed to access a particular ledger which, in turn, results in a difficulty for attacking and manipulating individual node’s ledgers [13].

#### 2.3.4. Trust

In a traditional centralized cloud and edge platform, trust and security are major issues to be addressed as before a transaction, nodes require trust between each other. In blockchain-based networks, cryptographic rules are the only ones upon which the nodes rely and transact [14].

#### 2.3.5. Tamper Proofing

Blockchain is designed in a manner where to attack and modify a particular block content, the intruder needs to subsequently alter the next blocks also. As the corresponding hash values get changed, such an action requires consensus from the maximum nodes in the network. The role of different nodes in a blockchain can be of three categories:

- Light node: It is responsible for storing a particular part of information observed in the system.
- Full node: It is responsible for storing all the information of the recorded transmission in the blockchain network.
- Forging or mining node: It is responsible for processing transactions, compiling the allowed transactions, and adding the block in the blockchain for further broadcast in the entire system.

### 2.4. Working Mechanism

The working mechanism of blockchain can be described by the following steps.

Step 1 When two users initiate a transaction based on their cryptographic key pair, all the other nodes are asked in the network to check the transaction’s validity based on the cryptographic public key and the corresponding signature which is a private key by nature.
Step 2 Upon confirmation received from neighbors, the transaction is forwarded to all the closely located nodes and is received after a certain period.
Step 3 Upon getting approval from the consensus, the transaction is added to the chain consisting of the other valid transactions and, in turn, gets ordered and packaged within a timestamp by the mining process.
Step 4 While the mining process is performed, the participating nodes have to undergo a computationally concentrated task depending on the protocol followed, such as Proof of Work. After completion of this protocol, the miner gets allowed to broadcast and add the newly created block to the chain.
Step 5 The blocks in the chain consist of mainly five parts, namely current block hash, previous block hash, timestamp, data and, index, where the index keeps track of the sequence of the blocks by assigning each block a particular number. The hash function is responsible for converting the block contents to exclusively allotted output, each having a static length [15]. The current block refers to the hash of the previous blocks always, which, in turn, invalidates all the next blocks when some hash value gets changed. This feature of the blockchain helps to lessen the chance of getting tampered by unauthorized users.

A comparison of this proposed approach with four existing related techniques is presented in Table 2.

## 3. Proposed Methodology

**Figure 2:**
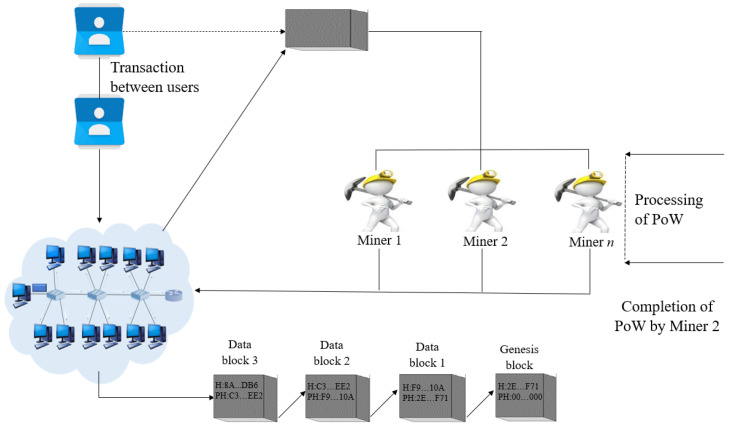
Workflow in the proposed system.

The design principles and workflow of this proposed scheme is introduced in this section as shown in Figure 2. A progressively decentralized blockchain-edge integrated framework has been proposed to increase the security and effective delivery of service in the edge platform. A novel privacy-preserving authentication system, inspired from [22], has been illustrated where there is no requirement of any third-party interrogation in registration or mutual authentication of the nodes involved in it.

### 3.1. Proposed Architecture

**Figure 3:**
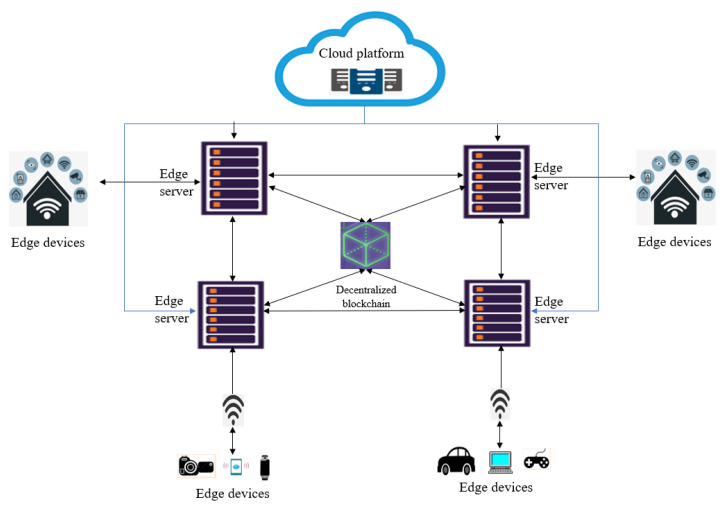
Architecture of this proposed model.

The architecture of this proposed model includes different components, i.e., edge servers, edge devices, and cloud servers having wired as well as wireless connections between them as shown in Figure 3. Access allowance of a user to an exact server depends on the validation result of all the service providers by consensus in that particular network. When a service provider gets recognized as an element of the decentralized blockchain platform, the related activities get accommodated by a layer created externally around it, which adapts the distributed ledger. This distributed ledger’s significant consequence helps track every validated user identity, predominantly using their public keys. This layer also tracks the sub-transactions between the edge device and service provider to have better transparency between all the network elements.

### 3.2. Registration, Login, and Authentication of Node

Each and every node having a decentralized blockchain scheme installed in it gets registered either as an edge device or as a service provider. After satisfying all the predetermined conditions, only if the node is recognized and hence is a genuine service provider, it involves a genesis node to trigger trailing nodes, i.e., edge devices for performing a successful transaction as shown in Figure 3. The functionality can be formulated as follows. Let *D* be a generator of an additive cyclic group $G_{1}$ and $G_{2}$, a group of cyclic multiplication. Let the bilinear map $e:G_{1}\times G_{1}\to G_{2}$ satisfy the following features, while *d* is the prime order of *G*1, *G*2 and $g=e\left(D,D\right)\in G_{2}$.

Bi-linearity: $\forall N,M\in G_{1}$ and ∀ random numbers $b,a\in Z_{p},e\left(bN,aM\right)=e\left(N,M\right)bA$. Computability: There exists an algorithm for computing e(M,N)∀M,N∈G1. Non-degeneracy: If 1 represents the identity element of G1, there exists M,N∈G1 where e(U,V)≠1. Three hash functions represented by $H_{i}:{\left\{0,1\right\}}^{*}\to {\left\{0,1\right\}}^{n}$ get (2) Please check if the comma should be the decimal point, like 0.1, if yes, please modify. generated where *i* = 1, 2, 3. A fuzzy extractor bio-cryptosystem has been implemented in this system to secure edge devices in this network which generates an input pair denoted by {α,β} for a user $U_{i}$ and the probabilistic generator function Gen(.). The public parameters created and stored at the service provider’s end to be forwarded to all the users demanding access include {$H_{1},H_{2},H_{3},D,d,e,G_{1},G_{2},Gen\left(.\right),Rep\left(.\right),\gamma$} and follow the steps described below.

Step 1: Upon installing the decentralized client, every user $U_{i}$ selects an identity $ID_{i}$ and forwards it to the corresponding service provider $SP_{j}$.

Step 2: Upon receiving the id, the service provider shares that with all the service providers to validate it. After completing the validation process by most of the nodes, the public parameters get shared with the user $U_{i}$ by the service provider.

Step 3: Whenever the user ${}_{i}$ receives pre-defined parameters, it selects $a\in Z_{p}$ and creates the private key as $S_{i}=a.ga.modd$ and the public key as $PK_{i}=S_{i}P$ and forwards them to the service provider immediately.

Step 4: The service provider adds the identity and public key to its chronology, also known as a record, and dispatches the narrative to all the connections and participating service providers to add it to the trail of the distributed ledger [22]. All the corresponding public keys and identities of the network $\left[\left(SP_{1},PK_{1}\right),\left(SP_{2},PK_{2}\right),\cdots ,\left(SP_{n},PK_{n}\right)\right]$ get shared with all the service providers in the network.

Step 5: The user $U_{i}$ generates $\left(\alpha_{1},\beta_{1}\right)=Gen\left(f_{i}\right)$ where $f_{i}$ is the biometric of user *i* and the following parameters get encrypted as

(1) $$Q_{i}=E_{H_{2}}(ID_{i}||PW_{i})\left[\left(SP_{1},PK_{1}\right),\left(SP_{2},PK_{2}\right),\left(SP_{3},PK_{3}\right),\cdots ,\left(SP_{J},PK_{J}\right)\right]$$

(2) $$q_{i}=E_{H_{1}}\left(\alpha_{i}\right)(ID_{i}||PW_{i}\left|\right|S_{i})$$

For encrypting the identities, public keys get generated using a user identity and private password known only to the user. So, the service provider record and public key cannot be extracted by someone without knowing $\left(ID_{i}||PW_{i}||f_{i}\right)$, and malicious attacks [17,23] in the system are prevented in this way.

### 3.3. Data Transmission between Service Providers and Nodes

After successfully validating nodes, they get added to the already connected nodes with the service provider. By creating a digital signature, they start to exchange and transmit information with the direct service provider or the service providers with which they are connected via their own service providers. Once a user is validated, it will not have to get registered again in the network even if they connect via some other service-delivering entity.

#### 3.3.1. Creation of Digital Signature

To authenticate and maintain integrity in user identity and data, a digital signature has been created in this proposed scheme using elliptic-curve cryptosystem-computing the transmitted message content between various nodes in the network. The process mainly includes generation and verification where generation takes *i*th user detail input in the form of $U_{i}:ID_{i},PK_{i}$, and *m*. Upon selecting anyone among the random numbers $a,b,c\in Z_{p}$, the computation takes place with the following:

(3) $$f=(H_{1}\left(m\right)\cdot ID_{i}\cdot P)$$

(4) $$d=\left(f\cdot PK_{i}\cdot a\right)\in Z_{p}$$

The created signature for the message *m* is denoted by $Sign_{i}=\left(f,d\right)$, the device of the recipient must undergo a verification stage where after receipt of the file, the receiver calculates *f*. Using the bilinearity rule, the signature validity gets tested and the transaction gets approved when it satisfies $e\left(Bd,aG\right)=e\left(f\cdot PK_{i},G\right)ba$.

#### 3.3.2. Initiation and Exchange of Transaction

The initiation and exchange of transaction can be described as follows.

Step 1. Upon receiving a request from a user for initiating a transaction by creating a file called $f_{t}$, the service provider instructs the user $U_{i}$ to choose a random number $v\in Z_{p}$ and calculates the exchanging transaction as

(5) $$H_{2}(b||PK_{i}||Sig_{i}||EPK_{j}\left(F_{t}\right)||PK_{j}\left||H\right)$$

Step 2. Upon receiving a transaction from the *i*th user $U_{i}$, the service provider checks its previous hash value and compares it with the last transaction performed between those particular two entities. If it is not matched, then the file is immediately driven out of the system.

Step 3. The transaction-requesting source gets verified by the public key. If it does not either correspond to digital signatures from both the end or the identities provided prior, then the transaction request immediately gets eliminated from the queue.

**Figure 4:**
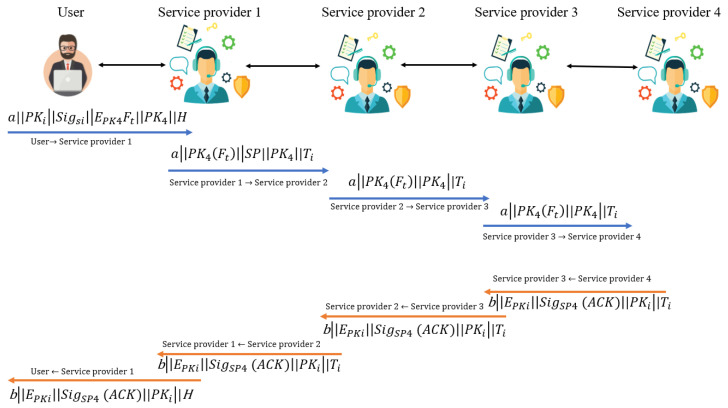
Transaction.

Step 4. The destination gets verified by the service provider to check whether the recipient is intended, or it is just forwarding the request. Based on it, the service provider confirms the signature’s authenticity in the first case, and the transaction gets decrypted with the public key. If the same was encrypted, then the file gets rejected in the second case, as it fails to satisfy the requirements of the system to get processed. When a validated user $U_{i}$ tries to connect with some service provider with which it is not directly connected, it connects through its connected servers as shown in Figure 4. Black double arrows indicate service exchange between user and service provider, here one user via three service providers.

Step 5. After taking delivery of a transaction, if a service provider realizes that the recipient is not the desired one, in that case, it packs the transaction again newly by deleting the hash value and the source. A random number gets generated after that for computation and the signature of a private key

(6) $$H_{1}(b||Sig_{S}P1||EP_{K}4\left(F_{t}\right)||PK_{4}\left|\right|T_{i})$$

The newly packed transaction gets forwarded to all its neighboring nodes.

Step 6. Upon receiving a transaction, the identity of the sender gets verified at first. If the sender’s signature is not genuine, then the transaction gets dropped, and if it is valid, then the next step taken is to verify the recipient’s identity. If the receiver is not the desired one, then the transaction gets transmitted to all the neighboring nodes and all of the service providers receive it within a short, stipulated time.

Step 7. The already forwarded transaction ids get eliminated from the network by service providers for avoiding network flood. The transactions which successfully get decrypted and the user validity gets verified, their ids get discarded, but if some transaction fails to do so, the transaction record is fetched again and tries to get authenticated for resource access. However, there is a time limit up to which a transaction is allowed to retry, otherwise, access gets revoked.

Step 8. Upon completion of reading the message which was carried by the transaction from the sender to the receiver, the recipient adds up the transaction to a dedicated block.

Step 9. Upon successful delivery of the transaction, the receiver sends an acknowledgment to the sender as

(7) $$H_{2}(b||EPK_{i}(Sig_{S}K4\left(ACK\right)||PK_{i}))$$

Before installing the BlockEdge client, the users are grouped using a clustering algorithm. Among *n* number of users, first, random *k* number of users are selected and the rest n−k number of users are represented by medoids based on the distance from the medoids. The clustering method looks as follows (Algorithm 1).
**Algorithm 1:** Clustering**Input:** K,C **Output:**
$C_{final}$ 1: function CLUSTERING(K,C) /*declare the center of the cluster*/ 2: RandomCenter[K]=V /*Initiate assigning clients*/ 3: for $C_{1}\in C$ and $V_{j}\in V$ do 4: if $cos\theta \left(C_{i},V_{j}\right)gtCos\theta \left(max\right)$ then 5: Add $C_{i}$ to the cluster of $V_{j}$ 6: end if 7: end for /*Modify the center of the cluster*/ 8: $C_{final}=C_{i}$ 9: Go to the next iteration 10: Return $C_{final}$ 11: End function


**Figure 5:**
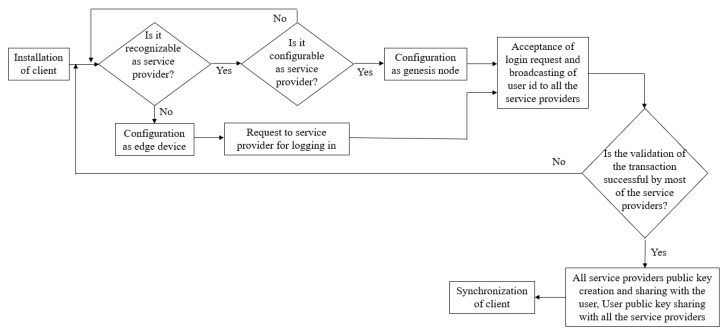
Work flow diagram of node initiation in the proposed approach.

For every point, the criterion function is computed and the point which corresponds to the minimum function value is selected as the updated medoid. The process repeats until the medoid points stop changing and reached the maximum predefined number of iteration. The workflow in initiation process of nodes is shown in Figure 5.

### 3.4. Cryptoanalysis

The proposed method’s capability of preventing the probable attacks originating from external as well as internal attackers can be analyzed by the following points:

i. The polynomial adversary monitors and controls unreliable transactions established on malicious links and, in turn, is able to modify, delete, replay, and reroute the transactions.
ii. The private key of any network object at any random time has increased entropy, so it cannot be predicted within a polynomial time.
iii. Every public network parameter is known to the polynomial adversary. It has the right to alter the hash values of ongoing transactions as well as the transactions to be added in the chain.
iv. The adversary polynomial is not capable of decrypting every ongoing or complete transaction of the network, it can only capture the leakage in information.

#### 3.4.1. User Confidentiality and Impersonation Attack

User record protection during a loss or theft is taken into account in this phase. Any kind of impersonation attack is attempted to be prevented in this proposed system. Suppose, the adversary intercepts every exchanged transaction in the system, as the hash value $E_{H_{1}}\left(\alpha_{i}\right)\left(UID_{i},P_{i},S_{i}\right)$ is encrypted with biometric *i*th user details such as secret key $K_{i}$, user id $UID_{i}$, password $P_{i}$, thus accessing the credentials would be a difficult job for it. For the retrieval of the service provider’s public key and identity details such as $\left(UID_{i},P_{i},S_{i}\right)$, at first, a biometric key α should be created using a reproductive deterministic function repr(.) consisting of β and $f_{i}$ as input. Thus, it is hard for the adversary to retrieve user details and impersonate the user in order to initiate an attack in this proposed system.

#### 3.4.2. User Non-Traceability and Secrecy

Fresh users in all the sessions only participate in transaction exchange for a random time being. That is why transaction linking is not possible within different sessions which, in turn, leads to non-traceable identity generation for users to prevent adversaries from tracking them in spite of a successful interception. Thus, this proposed design is secure enough for users interested to transact within the scheme.

#### 3.4.3. Impersonation Attack to Service Provider

To maintain confidentiality from both sides, the service provider’s identity is kept secret also, and it is transmitted only upon valid encryption. If the adversary captures a transaction between the server and some entity, it is still difficult for it to impersonate the service provider to do that, as it needs the digital signature designed by only the service provider’s private key. Additionally, the hash function referencing also prevents it from interacting or discussing with the nodes residing in the network.

#### 3.4.4. Man in the Middle and Replay Attack Scenario

Suppose some adversary tries to capture a transaction between two service providers or a service provider and a node. In that case, the system needs the hash referencing of the previously conducted transactions among the group; so, any initiatives with wrong hash values get automatically identified and treated accordingly. The timestamp also lets the servers know when a transaction replay attack is attempted. So, this proposed scheme is highly secure from these adverse scenarios.

## 4. Experimental Setup and Result Discussion

The experimental setup and the result is discussed in this section along with the performance analysis of the proposed approach with different parameters.

### 4.1. AVISPA-Based Cryptoanalysis

For security protocol-based model analysis, Automated Validation of Internet Security Protocols (AVISPA) is a useful tool where the protocols are mainly designed using High Level Protocol Specification Language (HLSPL). hlpsl2if is the translator which converts this high-level language to intermediate format (IF) having four backends, called Tree Automata-Based Protocol Analyzer (TABPA), Sat-based Model Checker (SATMC), CL-based ATtack SEarcher (CLATSE), On the Fly Model Checker (OFMC), which are able to read the high-level specifications in the IF. Among these four backends, OFMC is efficient to detect attacks and verify the protocols. Thus, it has been selected by us and the considered simulation goals have determined the simulation result.

Through the Dolev-yao channel the proposed scheme has been modeled in AVISPA, which includes an intruder to test the scheme’s maliciousness when a transaction is traversed in the established group. Any transactions and data dispatched to a genuine user(s) can be modified by intruder *i*. The simulation targets two major outcomes are:

i. Testing the Dolev-yao model.
ii. Testing the replay attack scenario.
iii. Execution capability checking of the non-trivial high-level protocol specification language.

**Figure 6:**
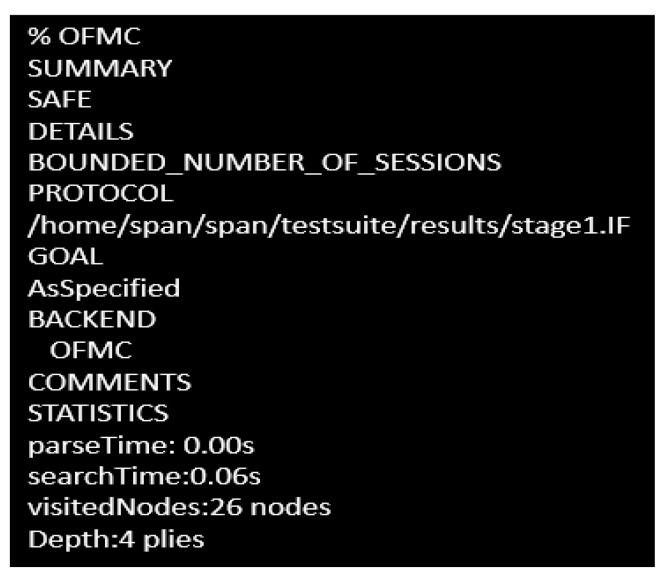
Simulation outcome in stage 1.

**Figure 7:**
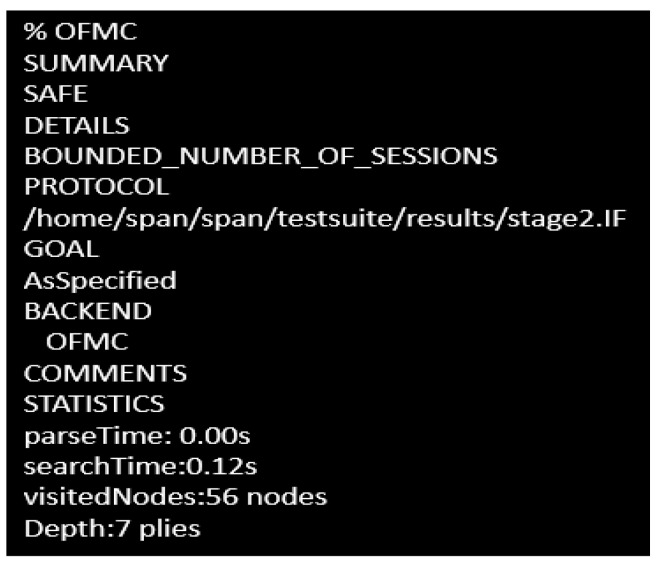
Simulation outcome in stage 2.

To check the Dolev-yao model, OFMC at first checks the probability of initiating a man-in-the-middle attack by the intruder. The experimental outcome has shown that the intruder has failed to initiate that attack, so the proposed scheme guarantees robustness as desired. In both stages, the intruder has been provided with the transaction information between different users in the network in both stages. The obtained outcome has proved this proposed approach to be an effective one against the active attack and passive attack as well. The simulation results for stage 1 and stage 2 are shown in Figure 6 and Figure 7, respectively.

### 4.2. NS2-Based Practical Simulation

The applicability of this proposed approach in the practical world has been tested using the NS2 2.35 simulator. Network protocols are simulated widely using event-driven simulation tools such as NS2. Three different situations in two different stages have been taken into account to check the applicability here. The impact of the system on different resources of the service providers each having a uniform number of mobile users in all three situations has been focused on, where the impact of computational resources on mobile users was neglected, as the considered metric values in all the simulation stages were virtually the same in that case.

The hash function has kept in length as 160 bits, randomly created number size as 128 bits, and the duration of authentication [24], digital signatures, and transaction ids as 32 bits long. Mobile users were consistently distributed among service providers in both stages in all three situations. The first stage is concerned with connecting a user to its corresponding service providers for computing and sending the transaction file and acknowledgment from the service providers to the user. The second stage is concerned with the exchange of transactions between the user and the intended service provider via the service providers to which the user is connected to. In the first stage of this experimental simulation, two transactions took place between *i*th user and *j*th service provider, where the first one was sending the user biometric to the service provider, and the second one was the acknowledgment from the service provider to the user. The transaction composition parameters were set as 554 and 364 bits, respectively. As Service provider 4 is not directly connected to the user, the intermediate Service Provider 1, Service Provider 2, and Service Provider 3 helped it to reach the destination by forwarding the transaction between the entities. The simulation parameters are discussed in Table 3.

The factors which were analyzed in two deployment platforms, i.e., service providers and mobile devices are energy consumption in mW, load in bps, and throughput in bps. The analysis has been performed depending on the calculation of the average of total packets sent, total packets received, packet size in bits, starting energy of *j*th service provider, starting energy of *i*th user, simulation time, and residual energy.

#### 4.2.1. Impact on Network Load

The average load in the network consisting of service providers and mobile nodes has been analyzed and calculated by

(8) $$Load=\frac{\begin{array}{c} (Packets\;sent\;+\;Packets\;received)\;\times \;Packet\;size \end{array}}{Simulation\;time}$$

In the stage 1 simulation, the service provider’s average load values in three different situations were 80.63 bps, 170.87 bps, and 225.96 bps, respectively. Since the network load increased with an increasing number of users on the service provider side with time, the second stage required three more service providers to meet the requirements of the user and the average load in the three situations became 47.98 bps, 69.56 bps, and 102.34 bps, respectively. As the source node had to involve three other service providers to forward the transaction to service provider 4, the network had to deal with a slight load increase but it was never a burden to the service providers at all, as they have large capacities and efficiency to perform the task.

#### 4.2.2. Impact on Network Throughput

The network throughput defined as transmitted bit per unit time has been calculated by

(9) $$Network\;throughput\;=\;\frac{\begin{array}{c} Packets\;received\;\times \;Packet\;size \end{array}}{Simulation\;time}$$

**Figure 8:**
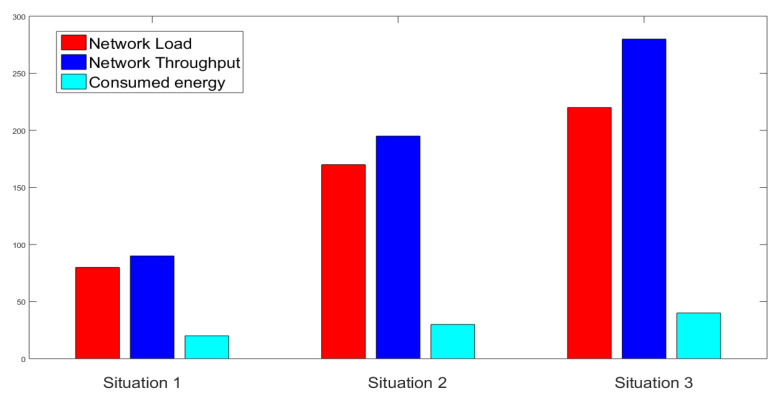
NS2 Simulation in Stage 1.

**Figure 9:**
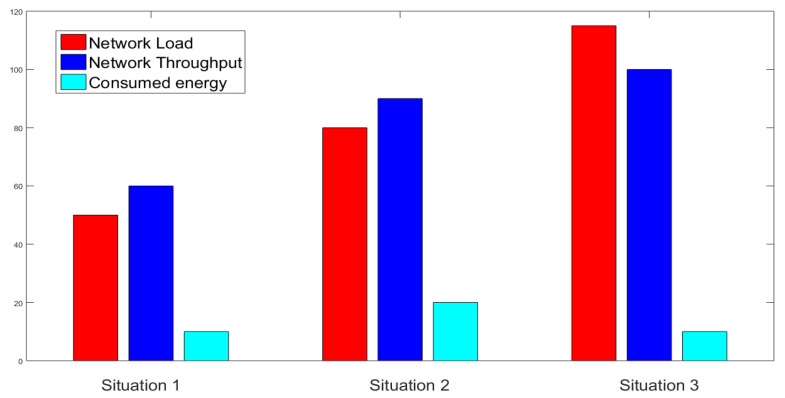
NS2 Simulation in Stage 2.

In the first stage, the average throughput has been obtained as 90.97 bps, 190.46 bps, and 280.42 bps, respectively, in situation 1, situation 2, and situation 3. The rise in mobile device users has resulted in an increased number of transactions in the system which, in turn, has obtained increased throughput in stage 2 as 145.41 bps, 223.98 bps, and 245.26 bps in the three situations, respectively, as displayed in Figure 8 and Figure 9.

#### 4.2.3. Impact on Consumed Energy

The impact of the proposed scheme on node energy consumption has been analyzed since many edge devices suffer from energy and resource restrictions. The consumed energy has been calculated by

(10) $$Consumed\;energy\;=\;\frac{\begin{array}{c} Initial\;energy-Residual\;energy \end{array}}{Simulation\;time}$$

The average energy consumption of service providers in stage 1 was 20.45 mW, 27.63 mW, and 37.84 mW, respectively, in the three situations. Later, the increase in network load led to increased energy consumption and thus, in the second stage, the consumed energy is 3.98 mW, 18.90 mW, and 11.14 mW, respectively, in situation 1, situation 2, and situation 3.

### 4.3. Performance Analysis

**Figure 10:**
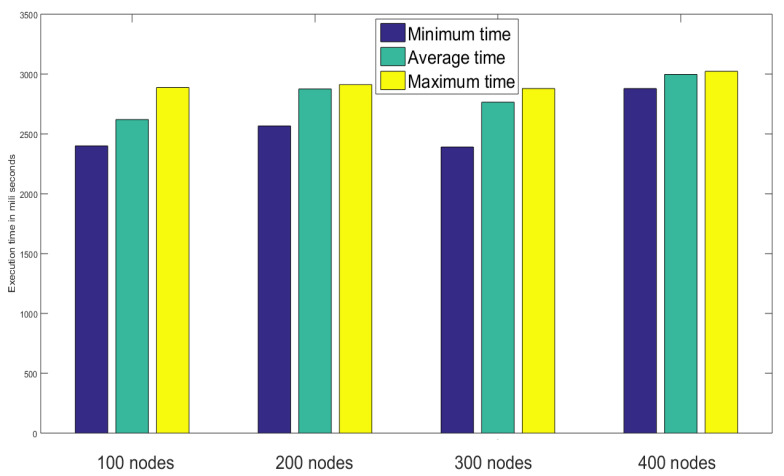
Performance analysis based on node creation and authentication time.

The performance and efficiency of the proposed method, in this article, have been evaluated by comparing them with [16,17] in terms of computational resources. Execution timing calculation has been done following [25]. Depending on PBC library [26], the base field in both the stages of the proposed approach are of order 512 bits in length and execution timings are of 150 bits in length. The time needed to execute and perform a multiplication of elliptic curve on $G_{1}$ denoted by $T_{mul}$ while $T_{par}$ denotes the execution time for an asymmetric bi-linear pairing operation, just like in [16]. The execution time for creating and authenticating nodes in the network has been analyzed by varying the number of nodes from 100 to 400 in Figure 10 where for each group among four groups having 100, 200, 300, and 400 nodes the minimum, average and maximum time have been recorded.

**Figure 11:**
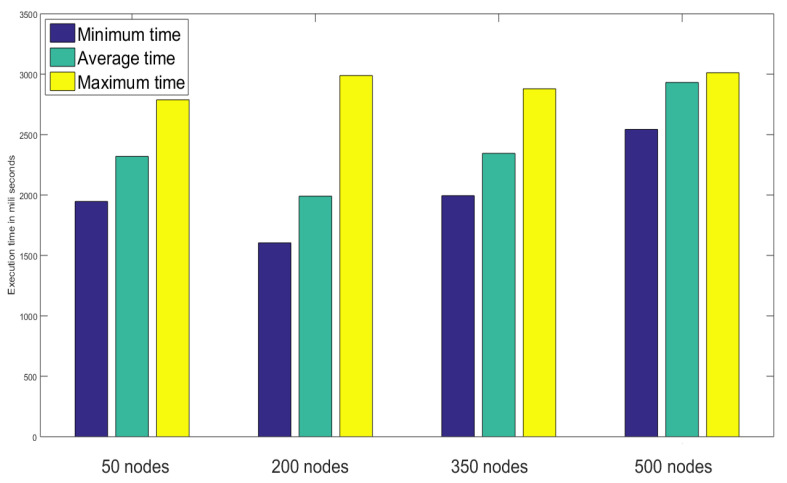
Performance analysis based on sensor record reading time.

The sensor record reading is a vital task in IoT platforms as it’s accuracy defines the whole system’s efficiency and effectivity as well. The execution time for recording and storing sensor data has been analyzed in terms of minimum, average, and maximum time like the last scenario in Figure 11, where the graph was steady and the overall capability of the system could be better evaluated if there was no network congestion.

**Figure 12:**
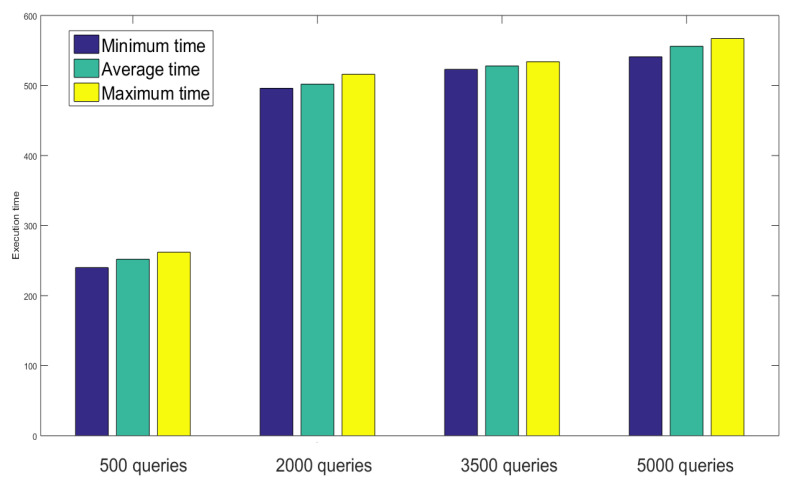
Performance analysis based on sensor query execution time.

During fetching the sensor records from the distributed ledger, the execution time has been analyzed for 500 to 5000 number of queries. It is clear from Figure 12 that number of records has a large impact on the network latency. The worst case was observed for 5000 queries as it took 541, 556, and 567 ms as a minimum, average, and minimum time to execute the operation.

**Figure 13:**
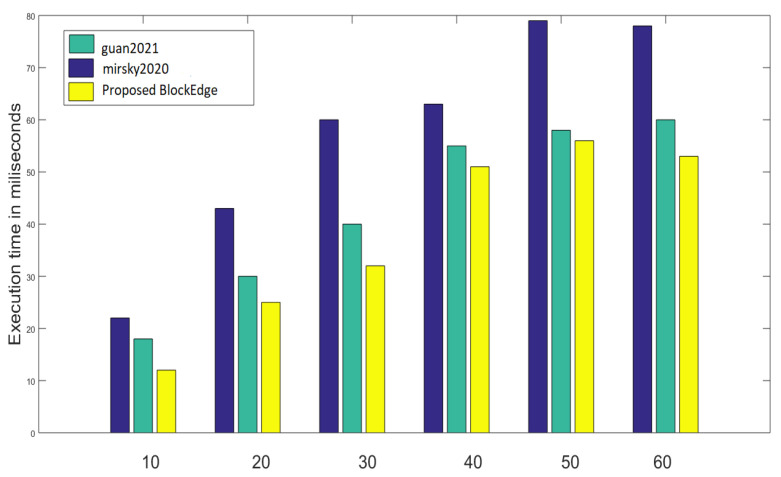
Performance analysis based on processing overhead [1,4].

The processing overhead of this proposed approach has been compared with [1,4] to evaluate the performance of BlockEdge, shown in Figure 13; where the blockchain network consisting of 60 blocks has been simulated for 70 s and 1030 transactions have been performed. This proposed approach has reduced the processing time overall by 34% which is really a novel contribution in the related domain.

Light computations: Bitwise XOR operation and cryptographic hash function have been omitted to deliver a comparison on equivalent parameters. The calculation of computational cost in the proposed approach for the mobile user as well as the service provider were performed analyzing the iPhone 6s and i5–4200M cost, respectively, as seen in Table 4. The computational cost of user and service provider in the proposed approach is denoted by $4T_{mul}\approx$ 27.3 ms and $4T_{mul}+2T_{par}\approx$ 4.8 ms, respectively. The computational costs in Odelu et al.’s approach [16] have been denoted by $6T_{mul}\approx$ 39.6 ms and $5T_{mul}+4T_{par}\approx$ 7.8 ms for user and service provider, respectively. The computational cost of Amin et al.’s [17] approach has been denoted by $5T_{mul}\approx$ 36.8 ms and $3T_{mul}+3T_{par}\approx$ 8.8 ms for user and service provider, respectively. This proposed method’s computational cost has been reduced due to the absence of a TTP unlike [16,17]. Besides that, the approach offered the same features of security incurring a lesser charge for the involved elements to execute the approach successfully as shown in Table 5. Security features of this proposed scheme were also analyzed and compared with a few similar approaches discussed in [16]. The approach in [16] consists of three entities, i.e., mobile user, service provider, and Smart Card Generator (SCG). This proposed approach integrated blockchain which, in turn, resulted in not involving some trusted third party in the scheme. Mobile users and service providers generated their public and secret keys.

## 5. Conclusions and Future Scope

The traditional edge platform authorization principles face a lot of challenges [27,28] including a single point of failure, scalability, etc. The introduction of blockchain in this framework reduces these issues for its decentralized and distributed nature, but it still suffers from some new structural challenges because of the various design principles [29]. In this paper, the challenges have been identified and discussed at first, and then, a novel scheme has been proposed incorporating the benefits from both operating principles. A decentralized design has been proposed for better privacy and security to be applied in different edge computing platforms for Industrial IoT and Industry 4.0/5.0. The robustness and cryptographic security have been tested using the AVISPA simulation tool for replay attacks and a man-in-the-middle attack scenario. Cost relative to computational resources has been used as the efficiency measuring tool of this proposed scheme in a practical simulation also, which, in turn, has proved the superior performance of the scheme compared with the existing ones in the concerned domain. The proposed method is well-suited for any user and serves its purpose efficiently. The system’s applicability can be tested for some other attack scenarios, such as brute force and Coppersmith’s attack in the future.

## Figures and Tables

**Table 1:** Comparison among different edge paradigms.

	Dew Computing	Fog Computing	Mobile Edge Computing	Mobile Cloud Computing
Purpose	Without using the features of cloud, provide service to customers in on-premise devices	To satisfy the requirements of delay-aware services and IoT geographic distribution	To reduce the latency, shift the cloud storage and computation from network to network edge	To improve the quality of service delivery, a delegation of computation and storage to edge devices
Deployment platform	User device	Edge and closer to edge devices	Network edge	Edge devices
Deploying infrastructure	On premise devices	Heterogeneous servers, switches, access points, routers, etc.	Heterogeneous servers, Various base stations, Radio access points, etc.	Heterogeneous servers, User devices, Various base stations, Radio access points, etc.
Ownership	Private and single owners	Private and single owners	Tele-communication organizations	Private and single owners
Target users	Common Internet users including mobile data user	Mobile data users	Mobile data users	Mobile data users

**Table 2:** Functionality comparison of the proposed block-edge approach with a few existing research works.

Ref. No.	TTP	User Trace Ability	User Privacy	Security in Mutual Authentication	Man in the Middle Attack Prevention	Re-Play Attack Prevention	Imper-Sonation Attack Prevention	Data Security
[16]	Yes	Yes	Yes	Yes	No	No	Yes	No
[17]	Yes	Yes	Yes	Yes	No	No	Yes	No
[18]	Yes	Yes	Yes	Yes	No	No	No	No
[19]	Yes	Yes	Yes	No	No	No	No	Yes
[20]	Yes	Yes	Yes	No	No	Yes	No	Yes
[21]	Yes	No	Yes	No	No	No	No	Yes
[22]	No	Yes	Yes	No	Yes	No	Yes	Yes
Block-Edge	No	Yes	Yes	Yes	Yes	Yes	Yes	Yes

**Table 3:** Factors considered for simulation in NS2.

Factor	Description	
Operating system	Ubuntu 20.04 LTS	
Simulation stage	Stage 1	Stage 2
Number of service providers	11 for situation 1, 2, 3	5 for situation 1, 2, 3
Number of service providers	11 for situation 1, 2, 3	5 for situation 1, 2, 3
	Situation 1: 15	Situation 1: 12
Number of customers	Situation 2: 25	Situation 2: 17
	Situation 3: 30	Situation 3: 33
User movement	6 m	
Primary energy of every service provider	600 J	
Primary energy of every user	700 J	
Simulation duration	1700 s	

**Table 4:** Computational terms in cryptographic operation in two frameworks.

	iPhone 6S	i5-4200M
$T_{mul}$	8.6 ms	0.9 ms
$T_{par}$	54.5 ms	2.1 ms

**Table 5:** Comparison in terms of computing cost.

Approach	Proposed in [17]	Proposed in [16]	Proposed in This Article
User	$5T_{mul}\approx$ 36.8 ms	$6T_{mul}\approx 39.6$ ms	$4T_{mul}\approx 27.3$ ms
Service Provider	$3T_{mul}+3T_{par}\approx$ 8.8 ms	$5T_{mul}+4T_{par}\approx$ 7.8 ms	$4T_{mul}+2T_{par}\approx$ 4.8 ms

## Data Availability

Not applicable.
